# Editorial: Natriuretic Peptides in Cardiovascular Pathophysiology

**DOI:** 10.3389/fphys.2022.890158

**Published:** 2022-05-18

**Authors:** Massimo Volpe, Speranza Rubattu

**Affiliations:** ^1^ Department of Clinical and Molecular Medicine, School of Medicine and Psychology, Sapienza University of Rome, Rome, Italy; ^2^ IRCCS Neuromed, Pozzilli, Italy

**Keywords:** natriuretic peptides, cardiovascular diseases, heart failure, prevention, therapy

The natriuretic peptides (NPs) family includes a class of hormones and their receptors needed for the physiological control of cardiovascular functions. Over the last 40 years, several experimental and clinical findings have clarified the fundamental contribution of these hormones to the physiological regulation of blood pressure and of cardiac, vascular, brain and renal functions. The present article collection includes a series of relevant papers discussing old and new concepts on the pathophysiological implications of NPs in cardiovascular diseases, the currently available NP-based drugs for the treatment of heart failure (HF), as well as the new molecules which will be soon tested in the clinical setting. Furthermore, an update is provided on a novel, molecular genetic-based approach aimed at the development of NP-based therapeutic applications in the treatment of major cardiovascular diseases (CVDs).

As shown by Cerrudo et al., increases of plasma atrial natriuretic peptide (ANP) without a concomitant increase of plasma B-type natriuretic peptide (BNP) indicate a state of atrial hemodynamic overload independently of ventricular hypertrophy. In fact, the ventricular re-expression of ANP is mainly induced in volume-overloaded deoxycorticosterone acetate (DOCA)-salt treated rats whereas BNP is induced in pressure-overloaded rats exposed to renovascular hypertension. The ANP/type A natriuretic peptide receptor (NPRA) axis is predominantly responsible for regulating the renal hemodynamic and Na + excretory responses to intravascular blood volume expansion. Notably, the effects of the NP systemic hormones are reinforced by a local renal NP system provided with the machinery for synthesis, action and degradation (Choi and Fernández). As strengthened in the article by Pandey, the ANP/NPRA axis provides cardiac protective mechanisms against maladaptive cardiac disorders, remodeling of CVDs and metabolic disorders. From a mechanistic point of view, an enhanced ANP-BNP/NPRA signaling protects the heart by inhibiting ventricular expression of NF-κB, a master regulator of proinflammatory cytokines. Within the endothelium, NPs exert several beneficial functions including anti-inflammatory and anti-thrombotic effects that support similar protective actions of the arm of the RAAS led by the ACE2-Ang (1-7)-MAS receptor. The combined beneficial vascular properties of NPs and ACE2-Ang (1-7)-MAS could reveal useful in several pathological conditions, even in the setting of the deleterious vascular consequences of the COVID-19 infection, as discussed in the article by Rubattu et al.

In an experimental model of cardio-renal syndrome, the aorto-caval fistula, an abundant expression of both corin and Proprotein Convertase Subtilisin/Kexin Type 6 (PCK6) is found in both cardiac and renal tissues as a possible compensatory mechanism able to enhance ANP/BNP actions and to counteract the development of cardiac hypertrophy, pulmonary congestion and renal dysfunction in this model of volume expansion (Khoury et al.).

Noteworthy, the review article by Miyoshi et al. shows evidence that the NP level assessed in umbilical cord blood and amniotic fluid may serve as a diagnostic biomarker of HF in fetuses with congenital heart disease and/or arrhythmia. This interesting result expands the common knowledge on the role of NPs as biomarkers for diagnosis of HF.

An intertissue communication between adipose and cardiac tissues has been documented in previous works. First, NPs play a lipolytic effect. Furthermore, the known inverse relationship between circulating BNP and body mass index (BMI), termed as the “natriuretic handicap,” is at least in part related to the increased expression of type C natriuretic peptide receptor (NPR-C) leading to enhanced degradation of BNP in adipose tissue. Moving from this evidence, the article by Egom develops the concept that the NPR-C pathway may play on its own a pathophysiological role, particularly in the context of obesity-related HF with preserved ejection fraction (HFpEF). In fact, it is worth mentioning that NPR-C plays biological functions other than the known role of clearance receptor. The signaling pathway underlying its biological properties may be the inhibition of adenylyl cyclase (AC) through a pertussis toxin–sensitive inhibitory G protein (Gi) or activation of phospholipase C (PLC) through Gi protein, therefore reducing adenylyl cyclase activity and intracellular cAMP levels. The author proposes the concept that the increased expression of NPR-C may in part explain the “obesity paradox” (that refers to the fact that obese patients with established HF tend to have better long-term prognosis than non-obese patients). Within the specific condition of obesity-related HFpEF, low NPR-C activity may promote cardiac fibrosis and remodeling, leading to diastolic dysfunction, the major cardiac functional deficit in HFpEF. Therefore, as pointed out by Egom, an enhanced NPR-C mRNA levels in various cardiometabolic disorders may represent a compensatory response to low NPR-C activity with the goal of re-establishing cardiometabolic function. The NPR-C pathway may represent a novel therapeutic target in cardiometabolic disorders, including but not limited to obesity and insulin resistance, in addition to HFpEF. Enhancing the NPR-C pathway may also represent an attractive therapeutic strategy to reduce body wasting, increase the ability to tolerate higher HF therapeutic doses of neurohumoral inhibitors, and improve HF outcomes.

In the context of hypertension and of anti-hypertensive therapy, the availability of agents enhancing the natriuretic biological functions has been intensively pursued over the last 2 decades. The recent design of MANP, a mimetic peptide, represents a promising strategy that fulfils the expectancy. In fact, MANP reduces blood pressure levels through the promotion of increased natriuresis, diuresis and aldosterone suppressing properties, similarly to native ANP. The first human trial confirmed the role of MANP as a valuable anti-hypertensive agent (Cannone and Burnett). The proANP 31-67 peptide, derived from the amino terminal fragment of NT-proANP, is another promising molecule. It shows cardiorenal protective actions in preclinical models and can be supported as a therapeutic strategy to counteract hypertensive and diabetic organ damage, renal diseases, obesity and HF (da Silva et al.).

Neprilysin/AT1R blockade (sacubitril/valsartan, S/V) is a novel therapeutic strategy introduced for the treatment of HF with reduced EF (HFrEF). As discussed by Gallo et al., the benefits of a S/V-based strategy have been demonstrated along most of the HF continuum, in which the neurohormonal dysfunction has a pivotal role in the development and progression of the disease. In fact, S/V appears to provide consistent benefits in a left ventricular EF (LVEF) range between 25 and 50%. This evidence suggests that HF patients with mid-range EF (HFmrEF) could be a reasonably successful target for S/V-based treatment, thus extending the current recommendations for this drug. As highlighted by the authors, a dichotomous vision of clinical presentations of HF, based on the LVEF values, should be dismissed in guidelines and trial designed clinical practice to define borders and boundaries of patient classification. Instead, the current evidence with S/V supports the notion that HF should be rather viewed as a continuous variable reflecting the whole spectrum of the properties of the LV.

Several possible therapeutic targets exist within the NP system beyond Neprilysin, all with the promise of improving HF treatment (Gidlof). In the specific, we know about the existence of epigenetic mechanisms actively regulating the NP system at multiple levels. Many of these mechanisms are active in the failing myocardium and they could potentially be exploited for therapeutic NP augmentation. They include several potential RNA targets such as miR-425 and miR-155. Furthermore, miR-100 and miR-143 could constitute potential RNA-based targets to achieve an increased level of circulating NPs. Currently, work is ongoing to elucidate the therapeutic benefit of NPPA-AS1 knock-down, a natural antisense transcript with potential regulatory capacity, in models of HFpEF and HFrEF. Finally, NPR-C silencing could result in increased circulating levels of ANP and reduced cardiac hypertrophy and fibrosis in HF.

Overall, this is an extremely interesting and expanding research field that needs many experimental and clinical studies addressed to meet the expectancies and ultimately to provide the next generation of NP-based therapy.

